# Stomatitis Materna: Report of Committee on Practical Medicine of Ill. State Medical Society for 1858: A Correction, Etc.

**Published:** 1859-03

**Authors:** J. N. Green

**Affiliations:** Stilesville, Indiana


ARTICLE II.

### STOMATITIS MATERNA.

REPORT OF COMMITTEE ON PRACTICAL MEDICINE, OF ILL. STATE MED. SOC., FOR 1858; A CORRECTION, ETC.

BY J. N. GREEN, M.D., OF STILESVILLE, INDIANA.

EDITORS JOURNAL: In reading the report of the Committee on Practical Medicine, of the Illinois State Medical Society, for 1858, as found in the January number of the *Chicago Medical Journal*, I find in that part of the report appropriated to the consideration of the treatment of *stomatitis materna* the following language: "Dr. Hutchinson, of Ohio, has found the bismuth an excellent remedy, not only in controlling the diarrhœa, etc., but also in improving the digestive power of the stomach." Now, it occurs to me that this is an incorrect attribution of the language used. The Dr. Hutchinson quoted is manifestly David Hutchinson, M.D., of Mooresville, Indiana. The quotation is from a prize essay which he wrote on stomatitis materna, for the Rhode Island State Medical Society, and which was published in the *American Journal of the Medical Sciences*, for October, 1857. The misapplication of the language to a Dr. Hutchinson of Ohio, is evidently an inadvertency on the part of the writer, as I am not aware that a physician of that name hailing from Ohio has ever written anything for publication on the subject of stomatitis materna.

Dr. Hutchinson, of Mooresville, Ind., has devoted very much careful and valuable attention and investigation to the subject of stomatitis materna, and it is just that he should have due credit for his liberal contributions to the heretofore, and even yet, very imperfect literature on that subject.

Let me here, also, call the attention of the committee to what I consider an important omission in their report. I allude to their silence on the use of chlorate of potassa in the treatment of the disease. If we are to believe the statements of numerous practitioners all over the West, who are almost daily writing and speaking in laudation of this remedy, it is entitled to credit as *the* remedy in stomatitis materna. And I am enabled to contribute my own limited experience in the use of the article, to the common stock on the subject.

The reasons which may have influenced the committee to entirely ignore, in their report, a remedy which has recently received so much prominence at the hands of the profession for its curative virtues in this disease, I am wholly unprepared to assign; but I am sure it is entitled to less *discourtesy* at their hands than it has received. I have prescribed it, with uniform success, in arresting the paroxysms of the disease. True, the number of cases in which I have used it have not been large, but the promptness with which it has relieved the suffering patient in these cases, has induced the belief in my mind that it is an invaluable remedy. I usually prescribe it in combination with pul. cinchonia. A cure is, of course, facilitated by the usual *et cetera*, which are not in the *programme*, but which are thrown in as adjuvants in the treatment, as well of this as other diseases.

The chlorate is chiefly useful as a hematogen, and, also, "from its catalytic action, by dissolving fibrin, and controlling the inflammatory process;" added to which is the salutary influence of the chlorine which it contains. Hence it will be perceived I do not claim for it the *specific* power over the disease which some have foolishly ascribed to it.

I will not close this article without speaking briefly of another remedy which comes to us with no mean pretensions to usefulness, in stomatitis materna. Its claims to our attention, in this connection, are not founded, however, on the success which has attended its administration; not on account of the trophies which it is enabled to bring with it to attest its power, for it is but just making its *debut* on the remedial stage; but because of its known *modus operandi* on the animal economy, and the success which has attended its imperfect trial in this and other diseases of analogous pathological conditions. I allude to the *glycerole* of the *hypophosphites*. Combining in an eminent degree the properties of a nutrient, hematogen, and nervous stimulant, it is peculiarly adapted to the cure of the disease. And, from what little personal knowledge I have of its utility, I am convinced a fair trial will establish for it such a reputation on an enduring basis.

There is still another point in this disease, of which I will speak, to which a profitable direction of scientific investigation may be given. It is to a "microscopic examination of the aphthæ, that occur in this disease, in order to determine whether it depends to any extent on fungi developed on the mucous membrane, as some have supposed." The settlement of this question would contribute important aid to the adoption of a more rational and successful practice in the treatment of the local symptoms than any which has been instituted.

